# 3D Immersive Patient Simulators and Their Impact on Learning Success: A Thematic Review

**DOI:** 10.2196/jmir.3492

**Published:** 2015-04-08

**Authors:** Robert Kleinert, Roger Wahba, De-Hua Chang, Patrick Plum, Arnulf H Hölscher, Dirk L Stippel

**Affiliations:** ^1^Department of General, Visceral and Cancer SurgeryUniversity of CologneCologneGermany; ^2^Department of General, Visceral and Cancer SurgeryCologneGermany; ^3^Department of RadiologyCologneGermany

**Keywords:** immersive patient simulators, Web-based learning, validity, immersion, procedural knowledge

## Abstract

**Background:**

Immersive patient simulators (IPSs) combine the simulation of virtual patients with a three-dimensional (3D) environment and, thus, allow an illusionary immersion into a synthetic world, similar to computer games. Playful learning in a 3D environment is motivating and allows repetitive training and internalization of medical workflows (ie, procedural knowledge) without compromising real patients. The impact of this innovative educational concept on learning success requires review of feasibility and validity.

**Objective:**

It was the aim of this paper to conduct a survey of all immersive patient simulators currently available. In addition, we address the question of whether the use of these simulators has an impact on knowledge gain by summarizing the existing validation studies.

**Methods:**

A systematic literature search via PubMed was performed using predefined inclusion criteria (ie, virtual worlds, focus on education of medical students, validation testing) to identify all available simulators. Validation testing was defined as the primary end point.

**Results:**

There are currently 13 immersive patient simulators available. Of these, 9 are Web-based simulators and represent feasibility studies. None of these simulators are used routinely for student education. The workstation-based simulators are commercially driven and show a higher quality in terms of graphical quality and/or data content. Out of the studies, 1 showed a positive correlation between simulated content and real content (ie, content validity). There was a positive correlation between the outcome of simulator training and alternative training methods (ie, concordance validity), and a positive coherence between measured outcome and future professional attitude and performance (ie, predictive validity).

**Conclusions:**

IPSs can promote learning and consolidation of procedural knowledge. The use of immersive patient simulators is still marginal, and technical and educational approaches are heterogeneous. Academic-driven IPSs could possibly enhance the content quality, improve the validity level, and make this educational concept accessible to all medical students.

## Introduction

**Figure 1:**
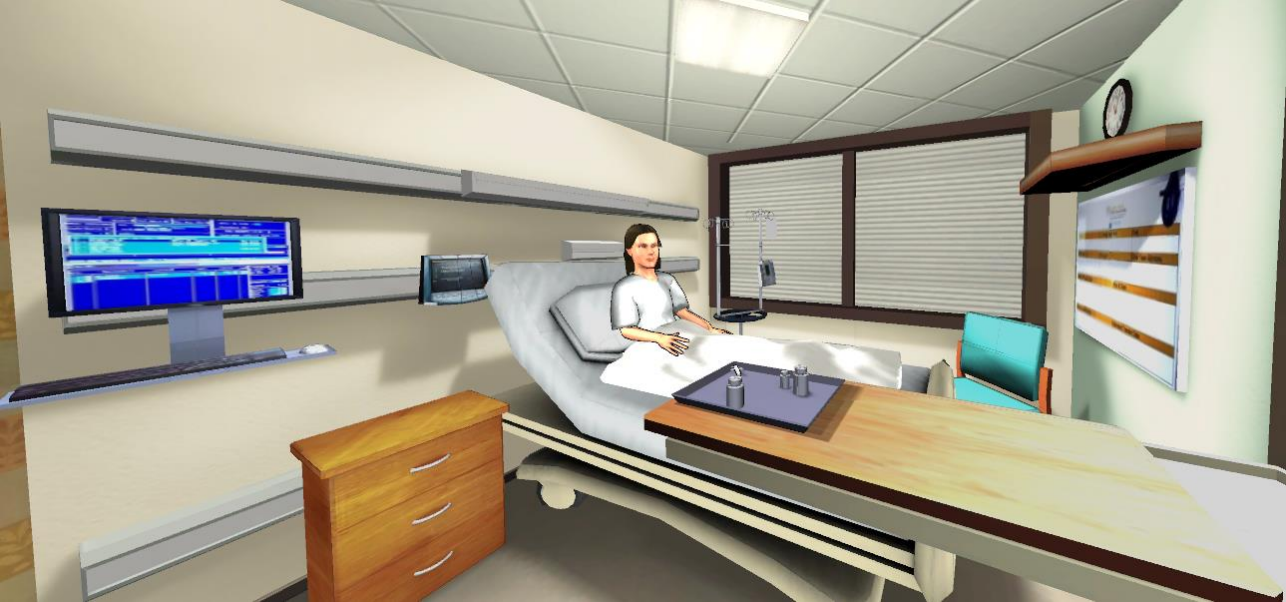
Example of a 3D virtual environment streamed via the Internet directly onto the learner’s computer (ie, Web-based) where the user can freely walk around and interact with the environment—authors’ project in collaboration with Clemson University, SC, USA.

One key factor of clinical education is the transfer of declarative knowledge (ie, “what to do”) into procedural knowledge (ie, “how to do”). It is performed most effectively in small groups accompanied by a medical teacher [1]. However, in the daily practice clinical instruction is often based on a traditional apprenticeship process and on the expectation that if a student spends enough time in a clinical environment he or she will eventually “get it”. Although this education is good clinical practice and known to be successful, it is impaired by increasing workload of hospital doctors, restrictive working time directives, and changes of students’ attitudes and expectations in the sense of the Generation Y [2]. Therefore, new educational strategies (eg, skills labs or mannequin simulators) are developed [3,4]. Virtual patient simulators go one step further as they allow case-based learning on personal computers. In a blended learning context they can have a positive impact on knowledge gain [5]. The available simulators vary greatly in realism and interaction grade, however it is questionable whether this factor affects learners’ outcome. Technological advances have allowed virtual patient simulators, such as immersive patient simulators (IPSs), with a high level of realism and interaction grade similar to computer games (ie, serious games). IPSs must not necessarily be installed on the user’s home computer (ie, workstation based). New technologies enable streaming of even complex programs via the Internet directly into the user’s browser (ie, Web-based). Web-based IPSs are characterized by representation of a three-dimensional (3D) virtual environment via the browser where users can freely interact in real time with their surroundings, and thus become part of the synthetic world, individually or in virtual teams (see Figure 1).

The user can playfully immerse himself/herself into the digital environment and faces the consequences of different decisions (ie, trial and error) without putting real patients at risk. By repetitive and playful training of medical procedures, procedural knowledge can be internalized and consolidated. Therefore, IPSs potentially allow time- and location-independent learning and an effective preparation for bedside teaching. However, evaluation of the impact of IPSs on knowledge gain is a demanding task, as gain in procedural experience can hardly be objectivized and is influenced by many educational factors. For medical teachers, information about validity and usability are essential parameters for a possible implementation of such simulators in the current medical curriculum. Furthermore, development of such a simulation is time- consuming and cost-intensive and only worthwhile when there is a proven effect on knowledge gain.

It was the aim of this paper to give a thematic review of the available immersive patient simulators in virtual worlds (VWs) and to evaluate whether the use of these simulators have an impact on knowledge gain by summarizing the existing validation studies.

## Methods

A systematic search of literature via PubMed of articles from 1986 to 2014 on IPSs was performed with a focus on education of medical students. IPSs were defined as digital environments that simulate medical workflows and show the characteristics of real-time simulation with free interaction in a 3D setting. As browser technology is developing fast, not only workstation-based simulators, but also Web-based simulators were included. Simulation of one-step procedures (ie, intubation, puncturing) were excluded. Search terms included a combination of “simulation,” “virtual reality,” or “virtual worlds,” and “education” *or* “training.” Peer-reviewed publications from 1986 until 2014 were included. Eligibility assessment was performed independently by two reviewers and disagreements between reviewers were resolved by consensus with a third reviewer. Titles and abstracts of all articles were screened with regard to relevance and consequently grouped—relevant articles and articles of unknown relevance were screened in full text. Furthermore, reference lists of relevant articles were searched for additional articles that were possibly not identified until then. Identified immersive patient simulators were classified by the field of application, technical parameters (ie, Web or workstation based, immersion grade, features), medical content (*low* ≤3 cases, *medium* = 4 to 8 cases, *high*≥9 cases), and existing validation studies.

Validation was assessed according to the consensus guidelines for validation of virtual reality surgical simulators [6]: (1) concordance (aka, face) validity refers to the degree of resemblance between simulator training and training in reality, (2) content validity refers to the degree to which simulated content covers the dimension of the construct it aims to educate, (3) construct (aka, contrast) validity describes the impact of existing knowledge on simulator performance, and (4) predictive validity describes the simulator impact on future performance applications [7,8]. Validation studies were further classified in accordance with the Cochrane Handbook for Systematic Reviews of Interventions. Studies were parameterized by methodical aspects (ie, study design, level of content), technical details of the IPS, end points, and study results. Validity of observational studies was assessed by using the methodological index for nonrandomized studies [9]. Literature review and data extraction was performed by two reviewers independently, and compared afterwards.

## Results

### Overview

**Figure 2:**
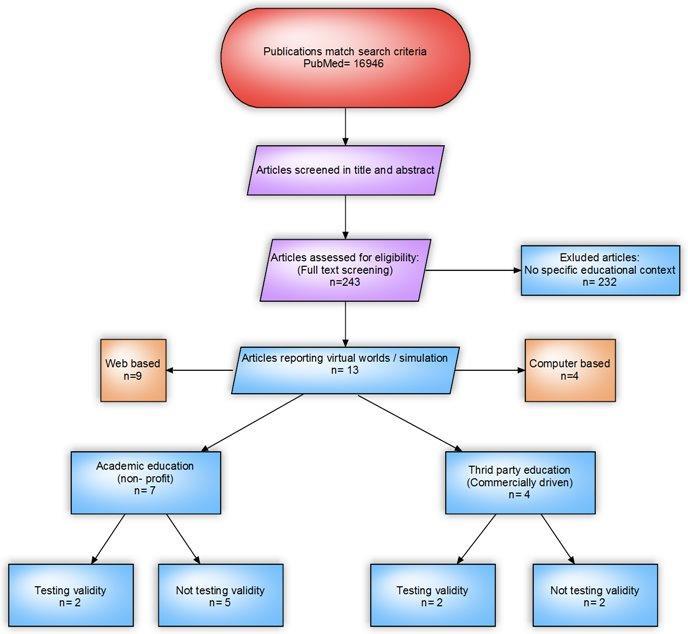
Search strategy for literature on virtual patient simulators.

The systematic literature search (Figure 2) identified 16,946 publications that matched the criteria.

A total of 13 publications were identified as relevant in terms of describing the use of virtual worlds in medical education (Table 1). Of these publications, 9 described IPSs that were Web-based and 4 described IPSs that were workstation based.

**Table 1:** List of available virtual patient simulators.

Virtual world	Type	Content	Immersion	Content level	Reference
VNEC	WB^a^	Neurological disorders	Medium	Medium	[10]
Play2Train	WB	Emergency medicine	Medium	Medium	[11]
MeRiTS	WB	Emergency medicine	Medium	Low	[12]
Second Health London	WB	Emergency medicine	Medium	Medium	[13]
CliniSpace	WB	Emergency medicine	Very high	High	[14]
Pulse	WB	Emergency medicine	High	High	[15]
3D Emergency Department	WB	Emergency medicine	Medium	High	[16]
Inmedea	WB	Various clinical cases	Low	High	[17]
Olive	WB	Emergency medicine	Medium	Low	[18]
Project TOUCH	CB^b^	Various clinical cases	High	Medium	[19]
Virtual Emergency Department	CB	Emergency medicine	High	High	[20]
TriageTrainer	CB	Triage	High	Low	[21]
Burn Center	CB	Burn wounds	High	Medium	[22]

^a^Web-based (WB).

^b^Computer-based (CB) (aka, workstation based).

### Web-Based Simulators

Out of 9 Web-based IPSs, 5 of them use the 3D technology of the widely used social network, Second Life (SL). All of these 5 IPSs were developed by university groups. None of these IPSs are routinely used in the education of medical students or medical staff, as they are technical feasibility projects with general medical content and focus on medical knowledge exchange [11]. However, they show the technical feasibility of providing medical education via virtual worlds [10]. A total of 3 of the Second Life-embedded virtual worlds were designed for team-based training in medical education—Second Health London is provided by the Imperial College London and was used in a pilot feasibility study including a first validation [13]. MeRiTS also offers team-based training in an SL environment, which was summarized in a feasibility study [12] without validation so far. Due to the technical specifications of Second Life, the graphical quality is not state of the art. This limitation potentially influences learners’ immersion, as graphical quality and presentation have an effect on immersion grade [23].

Web-based simulators are comparable to SL simulators, however, the main difference is the technical background. Web-based simulators use a proprietary 3D engine program, which makes development of 3D worlds more resource intensive, but enables more possibilities in graphical quality and custom program design. Of the 4 remaining Web-based IPSs, 1 was already presented in 2003 and uses a custom-made 3D world. Again, the key aspect of the simulator is a feasibility study, in this case particularly with regard to distance learning [19,24]. The 3 remaining Web-based IPSs were built by commercial software companies. Of these, 2 of them show a high 3D graphical quality and, thus, a high immersion grade. They offer team- and case-based training of acute clinical cases, with the main focus on emergency medicine [14]. The high quality is reflected by the elevated pricing of these simulations—more than US $5000 for a 1-year subscription. The medical content is high and custom-made virtual cases can be added for an additional fee. The CliniSpace IPS has already been used by Stanford University for emergency procedure training in the trauma room [16,20]. The remaining IPS, Inmedea, offers low immersion as it does not use a 3D engine, but consists of freeze-frames with drawn graphics. However, the amount of medical content in this simulator is very high, and custom cases can also be added when necessary. Although it is commercially driven, there are several universities that already use this simulator in a blended learning concept [25,26], and there are already first validation studies [17].

### Workstation-Based Simulators

Workstation-based IPSs need to be installed on a computer and offer high immersion with high-fidelity 3D graphics, as they are not limited by the technical handicaps of Internet broadcasting. Out of the 4 simulators, 3 of them are still undergoing feasibility studies, as they were not already validated for their effectiveness [22,27]. Although 1 of the simulators was introduced 10 years ago, it is neither routinely used in student education, nor has it been validated yet [19]. The remaining commercial VW environments by the University of Birmingham offer training in triage casualties in a simulated live exercise [21], which was part of the first validation study.

### Validation

The literature search uncovered 5 articles that included validation studies, however, no study assessed all levels of validity. One of the available VW simulators validated the coherence between simulated content and real content (ie, content validity) in training of emergency procedures [13]. The correlation between the outcome of simulator training and alternative training methods was proved in 2 virtual worlds [16,20] (ie, concordance validity)—in all 3 validation studies, simulator training was comparable to alternative training in terms of outcome. The coherence between measured outcome and future performance (ie, predictive validity) was validated in 2 simulators [21,17] and showed a positive correlation. The simulators and corresponding validation studies are summarized in Table 2.

**Table 2:** Overview of the validity level of all currently available virtual patient simulators.

Validity type and study		Number of participants	Virtual world	Reference
**Content validity**				
	Cohen et al, 2013	23	Second Health London	[13]
**Concordance validity**				
	LeRoy Heinrichs et al, 2008	30	3D Emergency Department	[16]
	Youngblood et al, 2008	30	Virtual Emergency Department	[20]
**Predictive validity**				
	Funke et al, 2012	116	Inmedea	[17]
	Knight et al, 2010	91	Triage Trainer	[21]

## Discussion

### Principal Findings

This paper gives an overview of the available IPSs and corresponding validation studies and, therefore, summarizes the current situation in learning in virtual worlds. Previous studies revealed that using virtual patient simulators can have a positive impact on learning success [5], whereas the effect of realism grade on knowledge gain is still under discussion. IPSs as a subgroup of virtual patient simulators offer high interaction grade and realism as in computer games (ie, serious games). However, we intentionally separated the group of immersive patient simulators from serious games, as the focus of these simulators lies more on knowledge transfer than on classical game elements. The potential of IPSs lies in the internalization of diagnostic and therapeutic procedures, such as resuscitation or diagnosis patterns. Ideally, the underlying procedures are already predefined to achieve performance uniformity, similarly to standard operating procedures in the clinical daily routine [28]. It is known that immersion plays a fundamental role in virtual reality simulators, as identification with the avatar influences motivation and improves learning success [23]. However, immersion grade is influenced by many factors [29] and, therefore, hard to verify. A study from 1999 revealed that “there were no statistical differences in presence or reality judgment between a high-impact workstation and a PC workstation” [30]. However, this study was conducted before the high-fidelity graphic era and the results are not entirely transferable, as the high-impact workstation (Silicon Graphics) from 1999 was still far away from the 3D capabilities of current standard personal computers, and realism grade was comparably poor. Furthermore, the majority of the students were not used to computer worlds and, thus, less susceptible to learning with 3D worlds. Newer studies revealed that there was a positive impact of high-fidelity visual presentation on degree of immersion [31] and even on learning performance [23]. For clinical teachers, the question arises whether IPSs can support the daily routine and have an effect on students’ future performance. Assessment of new educational concepts includes different forms of validity. Content validity describes the correlation between simulated and real content. There was a positive correlation between IPS learning and the standard clinical curriculum when training students on a well-defined procedure like triage training [21] or basic procedural workflow in emergency patterns [16,20]. In times of limited time resources in the daily clinical workflow, it is desirable that the clinical curriculum be effective both for teachers and students. IPSs can facilitate students’ preparation as they allow time- and location-independent learning at an individual learning pace and with repetitions, which ensure the attainment of a similar knowledge level by the participating students. Verification of predictive validity illustrates that there is an impact of learning with IPSs on future performance, and consequently enables successful preparation for hands-on training [17,21]. However, all current IPSs are used in the blended learning context, as postprocedural review of students’ performance immediately after training is known to be essential for an adequate knowledge gain [32]. Moreover, there is evidence that preexisting knowledge has a positive impact on simulator performance (ie, construct validity), although the group size of this study was low [13]. None of the articles assessed all forms of validity. As the studies are heterogeneous in medical content, the number of participants, and type of simulators, the initial question of whether the use of IPSs in the daily curriculum is beneficial in terms of learning success cannot be fully answered at this time. However, validation of single parameters revealed that IPSs can potentially support clinical teaching, although teachers must be aware of the limitations. IPSs are limited in terms of teaching declarative and procedural knowledge. Clinical education is not limited to teaching standard operating procedures, but is furthermore characterized by weighing clinical findings, evaluation of different hypotheses, and clinical experience. Therefore, IPSs are not intended to replace clinical teachers, but should support young students without relevant clinical experience. Regarding content, currently available virtual reality patient simulators range from small procedures, such as triage training, to complex procedures, such as emergency room protocols, up to teamwork with user-user interaction. The more complex the simulation, the more resources are needed. Therefore, it is not surprising that the complex simulations are commercially driven. Academic teachers can rent these simulators, making them more cost-effective, but it is questionable whether the development of a teaching method should be delegated. A highly immersive, multiuser, virtual reality simulator developed and supervised by an expert team of academic teachers would potentially allow, not only that the multiple users would train on one case (ie, team play), but also that one student would train on more than one patient at a time (ie, multitasking). These nontechnical skills can hardly be taught in reality, but are recognized as potential risk factors in high-risk environments like the emergency room or operating room [33]. It is the responsibility of universities and teaching hospitals to enable teaching methods that improve patient safety, reduce errors, and to further validate these new teaching methods.

### Conclusions

Immersive patient simulators can potentially promote learning and consolidation of procedural knowledge. Web-based simulators allow time- and location-independent learning at an individual pace. The use of immersive patient simulators is still marginal, and technical and educational approaches are heterogeneous. Academic-driven IPSs could possibly enhance the content quality, improve the validity level, and make this educational concept accessible to all medical students. The development and validation of such a simulator will be the subject of our future research.

## Abbreviations

3D: three-dimensional
IPS: immersive patient simulator
SL: Second Life
VW: virtual world

## References

[1] Sutkin G, Wagner E, Harris I, Schiffer R (2008). What makes a good clinical teacher in medicine? A review of the literature. Acad Med.

[2] Schlitzkus LL, Schenarts KD, Schenarts PJ (2010). Is your residency program ready for Generation Y?. J Surg Educ.

[3] Zendejas B, Brydges R, Wang AT, Cook DA (2013). Patient outcomes in simulation-based medical education: a systematic review. J Gen Intern Med.

[4] Gerdes B, Hassan I, Maschuw K, Schlosser K, Bartholomäus J, Neubert T, Schwedhelm B, Petrikowski-Schneider I, Wissner W, Schönert M, Rothmund M (2006). [Instituting a surgical skills lab at a training hospital]. [Article in German]. Chirurg.

[5] Cook DA, Erwin PJ, Triola MM (2010). Computerized virtual patients in health professions education: a systematic review and meta-analysis. Acad Med.

[6] Carter FJ, Schijven MP, Aggarwal R, Grantcharov T, Francis NK, Hanna GB, Jakimowicz JJ, Work Group for Evaluation and Implementation of Simulators and Skills Training Programmes (2005). Consensus guidelines for validation of virtual reality surgical simulators. Surg Endosc.

[7] Graafland M, Schraagen JM, Schijven MP (2012). Systematic review of serious games for medical education and surgical skills training. Br J Surg.

[8] Gallagher AG, Ritter EM, Satava RM (2003). Fundamental principles of validation, and reliability: rigorous science for the assessment of surgical education and training. Surg Endosc.

[9] Slim K, Nini E, Forestier D, Kwiatkowski F, Panis Y, Chipponi J (2003). Methodological index for non-randomized studies (minors): development and validation of a new instrument. ANZ J Surg.

[10] Boulos MN, Hetherington L, Wheeler S (2007). Second Life: an overview of the potential of 3-D virtual worlds in medical and health education. Health Info Libr J.

[11] Kamel Boulos MN, Ramloll R, Jones R, Toth-Cohen S (2008). Web 3D for public, environmental and occupational health: early examples from second life. Int J Environ Res Public Health.

[12] Chodos D, Stroulia E, King S (2011). MeRiTS: simulation-based training for healthcare professionals. Stud Health Technol Inform.

[13] Cohen D, Sevdalis N, Patel V, Taylor M, Lee H, Vokes M, Heys M, Taylor D, Batrick N, Darzi A (2013). Tactical and operational response to major incidents: feasibility and reliability of skills assessment using novel virtual environments. Resuscitation.

[14] Dev P, Heinrichs WL, Youngblood P (2011). CliniSpace: a multiperson 3D online immersive training environment accessible through a browser. Stud Health Technol Inform.

[15] Creutzfeldt J, Hedman L, Heinrichs L, Youngblood P, Felländer-Tsai L (2013). Cardiopulmonary resuscitation training in high school using avatars in virtual worlds: an international feasibility study. J Med Internet Res.

[16] LeRoy Heinrichs W, Youngblood P, Harter PM, Dev P (2008). Simulation for team training and assessment: case studies of online training with virtual worlds. World J Surg.

[17] Funke K, Bonrath E, Mardin WA, Becker JC, Haier J, Senninger N, Vowinkel T, Hoelzen JP, Mees ST (2013). Blended learning in surgery using the Inmedea Simulator. Langenbecks Arch Surg.

[18] Creutzfeldt J, Hedman L, Medin C, Heinrichs WL, Felländer-Tsai L (2010). Exploring virtual worlds for scenario-based repeated team training of cardiopulmonary resuscitation in medical students. J Med Internet Res.

[19] Caudell TP, Summers KL, Holten Ji, Hakamata T, Mowafi M, Jacobs J, Lozanoff BK, Lozanoff S, Wilks D, Keep MF, Saiki S, Alverson D (2003). Virtual patient simulator for distributed collaborative medical education. Anat Rec B New Anat.

[20] Youngblood P, Harter PM, Srivastava S, Moffett S, Heinrichs WL, Dev P (2008). Design, development, and evaluation of an online virtual emergency department for training trauma teams. Simul Healthc.

[21] Knight JF, Carley S, Tregunna B, Jarvis S, Smithies R, de Freitas S, Dunwell I, Mackway-Jones K (2010). Serious gaming technology in major incident triage training: a pragmatic controlled trial. Resuscitation.

[22] Kurenov SN, Cance WW, Noel B, Mozingo DW (2009). Game-based mass casualty burn training. Stud Health Technol Inform.

[23] Gutiérrez F, Pierce J, Vergara VM, Coulter R, Saland L, Caudell TP, Goldsmith TE, Alverson DC (2007). The effect of degree of immersion upon learning performance in virtual reality simulations for medical education. Stud Health Technol Inform.

[24] Alverson DC, Saiki SM, Kalishman S, Lindberg M, Mennin S, Mines J, Serna L, Summers K, Jacobs J, Lozanoff S, Lozanoff B, Saland L, Mitchell S, Umland B, Greene G, Buchanan HS, Keep M, Wilks D, Wax DS, Coulter R, Goldsmith TE, Caudell TP (2008). Medical students learn over distance using virtual reality simulation. Simul Healthc.

[25] Wünschel M, Leichtle U, Wülker N, Kluba T (2010). Using a web-based orthopaedic clinic in the curricular teaching of a German university hospital: analysis of learning effect, student usage and reception. Int J Med Inform.

[26] Horstmann M, Renninger M, Hennenlotter J, Horstmann CC, Stenzl A (2009). Blended E-learning in a Web-based virtual hospital: a useful tool for undergraduate education in urology. Educ Health (Abingdon).

[27] Lee CH, Liu A, Del Castillo S, Bowyer M, Alverson D, Muniz G, Caudell TP (2007). Towards an immersive virtual environment for medical team training. Stud Health Technol Inform.

[28] Papakonstantinou D, Poulymenopoulou M, Malamateniou F, Vassilacopoulos G (2013). Enabling the use of enhanced medical SOPs by an mLearning training solution. Stud Health Technol Inform.

[29] Kuhlen TW, Hentschel B (2014). Quo vadis CAVE: does immersive visualization still matter?. IEEE Comput Graph Appl.

[30] Botella C, Rey A, Perpiñá C, Baños R, Alcañiz M, Garcia-Palacios A, Villa H, Alozano J (1999). Differences on presence and reality judgment using a high impact workstation and a PC workstation. Cyberpsychol Behav.

[31] Huerta R (2012). Dissertations & Theses, The University of Texas - Pan American.

[32] Welke TM, LeBlanc VR, Savoldelli GL, Joo HS, Chandra DB, Crabtree NA, Naik VN (2009). Personalized oral debriefing versus standardized multimedia instruction after patient crisis simulation. Anesth Analg.

[33] Catchpole K, Mishra A, Handa A, McCulloch P (2008). Teamwork and error in the operating room: analysis of skills and roles. Ann Surg.

